# Poor Sleep Increases Next Day Pain, Particularly for Older Adults With Mild Cognitive Impairment

**DOI:** 10.1093/geroni/igaa057.1699

**Published:** 2020-12-16

**Authors:** Xiaopeng Ji, Mary Bowen, Mari Griffieon

**Affiliations:** University of Delaware, Newark, Delaware, United States

## Abstract

Sleep studies examine how pain is associated with poor sleep. However, emergent research suggests poor sleep increases pain and may interfere with activities of daily living (ADL) among older adults. This study will examine how poor sleep may affect next-day pain interference and how this relationship may vary by cognitive function. Ten community-dwelling older adults with lower extremity chronic pain wore an Actigraph GT9X Link for 7 days to measure poor sleep and next-day pain interference (Brief Pain Inventory; BPI). Multi-level mixed models accounted for intra-individual changes in sleep and pain interference and controlled for age, mild cognitive impairment (MCI) and depressive symptoms. Poor sleep among older adults with MCI (14 total observations) was also explored. Across 79 observations, increased number of awakenings (β=0.03; p≤ 0.05) and movement index scores (β=0.08; p≤ 0.05) were associated with increased next-day pain interference. In exploratory analyses, MCI intensified relationships between sleep efficiency (β=-0.10; p≤ 0.05), increased awakenings after sleep onset (β=0.01; p≤ 0.05) and increased length of sleep awakenings (β=0.39; p≤ 0.01) on next-day pain interference. This study’s findings suggest poor sleep is associated with next-day pain interference and the ability to perform ADL. Older adults with MCI may be at an increased risk for poor sleep and pain-related interference in ADL. Interventions designed to moderate the association between poor sleep and pain in general and for adults with MCI in particular may be warranted.

